# Supplementary material for: Dysregulated Cell–Cell Communication Characterizes Pulmonary Fibrosis
Source: Cells. 2022 Oct 21;11(20):3319. doi: 10.3390/cells11203319 (PMC9600037; doi:10.3390/cells11203319)
Supplement: Supplementary file 1 [file cells-11-03319-s001.zip › cells-1849196 supplementary publish update.pdf]

Supplementary Materials

Table S1. Demographic details of the Vanderbilt dataset (GSE135893).

	Control (10)	IPF (12)	*p* Value
Age (range)	35.2 (17–54)	65.17 (56–74)	1.135×10^{-5} (Students' *t*)
Sex (M/F/unknown)	7/2/1	7/5/0	0.6424 (Fisher)
Race (white/nonwhite)	6/1/3	9/3/0	0.9999 (Fisher)
Tobacco (Y/N/unknown)	7/1/2	6/6/0	0.1577 (Fisher)

Table S2. Demographic details of the Colorado dataset (GSE161685).

ID	1097743	1097858	1058227	1097691
Diagnosis	Control	Control	IPF	IPF
Genotype	GG	TT	GG	TT
Age	64	64	65	64
Sex	male	male	male	male
Race	white	white	white	white
Tobacco	unknown	unknown	unknown	unknown

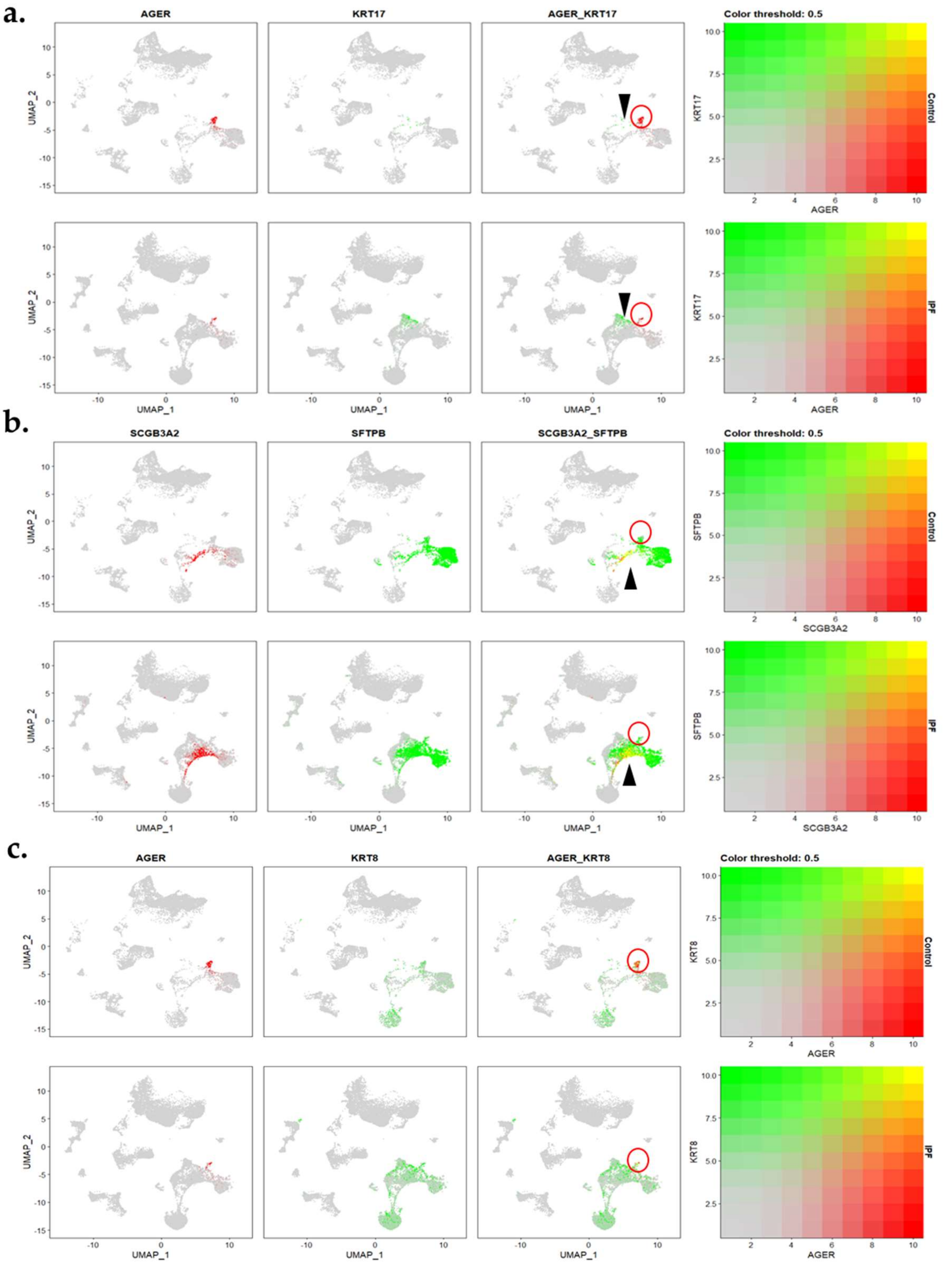

Figure S1. Identity of AECI cells used in these studies. The analyzed AECI cells were *AGER-*, *SFTPB-*, and *KRT8*-positive, and *KRT17-*, *KRT19-*, and *SCGB3A2*-negative, corresponding with fully differentiated cells. (**a.**) Minimal overlap with *AGER*+ AECI (red, circle) and "aberrant basaloid" cells as defined by *KRT17* positivity (green, arrowhead). (**b.**) Minimal overlap between *SCGB3A2*+ (red), *SFTPB*+ (green) pre-alveolar transitional cells (PATS, arrowhead), and AECI (circle). (**c.**) Overlap between *AGER*+ AECI (red, circle) and *KRT8*+ (green), a marker of impaired alverolar differentiation. Degree of overlap within cells for the two parameters is represented by blending of the two color parameters (red and green), shifting to yellow with high intensity.
